# Shame, self-disgust, and envy: An experimental study on negative emotional response in borderline personality disorder during the confrontation with the own face

**DOI:** 10.3389/fpsyt.2023.1082785

**Published:** 2023-03-08

**Authors:** Miriam Biermann, Anna Schulze, Ruben Vonderlin, Martin Bohus, Lisa Lyssenko, Stefanie Lis

**Affiliations:** ^1^Department of Psychiatric and Psychosomatic Medicine, Central Institute of Mental Health, Medical Faculty Mannheim, Heidelberg University, Mannheim, Germany; ^2^Department of Clinical Psychology, Central Institute of Mental Health, Medical Faculty Mannheim, Heidelberg University, Mannheim, Germany; ^3^Department of Clinical Psychology, Ruhr University Bochum, Bochum, Germany; ^4^McLean Hospital, Harvard Medical School, Boston, MA, United States; ^5^Department of Public Health, Freiburg University of Education, Freiburg, Germany

**Keywords:** borderline personality disorder, disgust, envy, experiment, self-conscious emotions, self-referential processing, shame

## Abstract

**Background:**

A markedly negative self-image and pervasive shame proneness have consistently been associated with borderline personality disorder (BPD). The present experimental study investigated the intensity of negative emotional responses with a focus on shame in BPD compared to healthy control persons (HCs) during an experimental paradigm promoting self-awareness, self-reflection, and self-evaluation. Furthermore, the relationship between levels of state shame during the experiment and shame proneness in BPD compared to HCs was examined.

**Methods:**

A sample of 62 individuals with BPD and 47 HCs participated in the study. During the experimental paradigm, participants were presented with photos of (i) the own face, (ii) the face of a well-known person, and (iii) of an unknown person. They were asked to describe positive facets of these faces. Participants rated the intensity of negative emotions induced by the experimental task as well the pleasantness of the presented faces. Shame-proneness was assessed using the Test of the Self-Conscious Affect (TOSCA-3).

**Results:**

Individuals with BPD experienced significantly higher levels of negative emotions than HCs both before and during the experimental task. While HC participants responded to their own face particularly with an increase in shame compared to the other-referential condition, the BPD patients responded above all with a strong increase of disgust. Furthermore, the confrontation with an unknown or well-known face resulted in a strong increase of envy in BPD compared to HC. Individuals with BPD reported higher levels of shame-proneness than HCs. Higher levels of shame-proneness were related to higher levels of state shame during the experiment across all participants.

**Conclusion:**

Our study is the first experimental study on negative emotional responses and its relationship to shame proneness in BPD compared to HC using the own face as a cue promoting self-awareness, self-reflection, and self-evaluation. Our data confirm a prominent role of shame when describing positive features of the own face, but they emphasize also disgust and envy as distinct emotional experience characterizing individuals with BPD when being confronted with the self.

## 1. Introduction

One of the core symptom domains in borderline personality disorder (BPD) are dysfunctions in emotion processing with a predominantly negative affect and impairments in emotion regulation (1). These impairments are part of etiological models such as the biosocial model of Linehan (2). This model assumes that an interaction between a high sensitivity to emotions as an example for a genetic factor and psychosocial factors such as adverse childhood experiences underlies the pathogenesis of BPD. In line, impairments in emotion regulation form the basis for disorder-specific therapeutic approaches such as the dialectic behavioral therapy (DBT; 3). Many conceptualizations suggest that there are emotions specifically linked to the processing of self-related information (4, 5). These so-called self-conscious emotions comprise, for example, shame and guilt, but also self-contempt or self-disgust (e.g., 4–7). Previous studies suggest that negative self-conscious emotions are particularly important in BPD (e.g., 8–10) with a special importance of shame (e.g., 11, 12). These studies have primarily used self-report questionnaires in which individuals have to imagine their emotional responses to theoretical scenarios. Findings support a higher proneness to shame in BPD. However, studies are missing that investigate whether increased levels of shame are only part of the overall increased level of negative affect in BPD or whether they are distinctively exaggerated responses evoked by specific contextual or internal factors. In the current study, we aim to contribute to the understanding of self-conscious emotions in BPD by investigating negative emotional responses during an experimental paradigm in BPD and healthy individuals and the association to the individuals’ shame proneness.

Shame is a cognitive affective construct, comprising negative judgments of the self (e.g., 13–15). Alongside other emotions such as humiliation, embarrassment and guilt, shame is a self-conscious emotion as it requires self-awareness, complex self-representations, self-reflection, and self-evaluation (4, 16, 17). Shame arises after one has failed to meet social or own standards and norms regarding what is appropriate and desirable (18, 19). It signals an actual or likely threat to self-esteem, social status, or acceptance. It has a potentially disturbing influence on the self-esteem and gives rise to feelings of worthlessness and inferiority (16, 20, 21). Shame motivates people to withdraw and isolate themselves from other people in order to either hide their inferiority, or to appease their social group by showing awareness of one’s norm-violating behavior and willingness to conform to group standards (e.g., 13, 22–24). Shame-proneness is the trait-like tendency to experience shame across a range of socially relevant situations stemming from internal, global, and stable attributions of negative events to the self (4). It is distinguished from “state shame” that is a transitory affective state restricted to a moment in time (11). Particularly in the context of shame as a target for therapeutic interventions, increased trait or state shame has different implications for psychotherapy that is, for example, changing a general stable attitude toward oneself or changing an automatic acute shame reaction arising fast as a reflex to specific triggers.

Shame is a self-conscious emotion of trans-diagnostic relevance. Increased levels of shame have been related to various mental disorders including social phobia, major depression, substance abuse, eating disorders, body dysmorphic disorder, psychosis, and posttraumatic stress disorder (24, 25). Studies contrasting different mental disorders suggest that both shame proneness and state shame are particularly central to the psychopathology of BPD (26, 27).

With regard to explicit shame proneness, individuals with BPD reported higher levels of shame compared to healthy individuals and other clinical samples such as major depression, social phobia, attention-deficit/hyperactivity disorder, and narcissistic personality disorder (11, 12, 28–31). However, higher levels of implicit shame-proneness, as measured with an Implicit Association Test, compared with anxiety-proneness, could not be consistently linked to BPD (11, 30). These findings point to differences between explicit and implicit measures of shame proneness, that is, when individuals evaluate their tendency to experience shame or to select a shame-led tendency to act in questionnaires (e.g., TOSCA, SHAME; 32, 33) in contrast to when shame proneness is inferred from performance data such as error rates or reaction times without the participants’ awareness of their behavior.

With regard to state shame, the findings are less clear and seem to be influenced by the measurement instruments as well as contextual factors such as the cues used to trigger a shame response. Results from cross-sectional studies assessing state shame with self-report questionnaires suggest elevated levels of state shame in BPD compared to healthy individuals and individuals with social phobia or narcissistic personality disorder (10, 11, 30). This particular relevance of state shame in BPD is supported by a previous study using experience sampling method (ESM): dynamics of high instability, interpersonal reactivity, and a prolonged return to baseline levels in guilt and shame after real live interpersonal challenges were specific to BPD compared with bipolar disorder (BD), major depressive disorder (MDD), and healthy control individuals (HC) even after controlling for co-occurrence of current MDD or BD in the BPD group (34). While Gadassi et al. (35) found an increase in shame following the experience of social proximity both in individuals with BPD and avoidant personality disorder during an ESM study, their findings revealed also simultaneously an increase of anger specifically in the BPD group. While these results on state shame in studies using self-report questionnaires and ESM revealed consistently stronger shame responses in BPD, experimental studies of state shame in BPD show mixed findings: Gratz et al. (36) investigated emotional reactivity and recovery in outpatients with BPD (*N* = 17) and outpatients without a personality disorder (non-PD; *N* = 18) following an experimental stress induction of anxiety, irritability, hostility, and shame. They examined the effects of two laboratory stressors, contrasting a negative evaluation of the participants with the Paced Auditory Serial Addition Task (PASAT) as a non-social stressor. The PASAT is an empirically supported laboratory stressor shown to induce emotional distress in the form of anxiety, frustration, and irritability (37–39). Individuals with BPD exhibited higher emotional reactivity and—as a result of the strength of their emotional reactions—a slower return to baseline levels of emotional arousal than non-PD. These changes were specific to shame and not seen for other emotions. Moreover, these effects were dependent on the particular stressor, inducing emotional distress only for the social but not for the non-social stressor. In contrast, Scheel et al. (40) found elevated baseline levels of shame in a group of individuals with BPD (*N* = 25) compared with MDD (*N* = 25) and healthy control persons (HC; *N* = 23). However, when asking participants to take either the perspective of a protagonist of a scenario describing a shameful job interview or of a scenario describing a person’s morning routine with neutral emotional content, results revealed no differences in the intensity of shame or return to baseline of shame in the BPD group compared with the MDD and HC group. Similarly to the ESM study by Gadassi et al. (35), Scheel et al. (40) found instead a prolonged anger reaction after completing the shame induction exercise.

Taking together, previous studies have focused either on shame-proneness or state shame in BPD. To our knowledge, there are no studies investigating whether shame proneness is related to state shame or specific shame triggers.

In sum, studies consistently suggest a particularly high shame proneness in BPD compared with other mental disorders. In contrast to cross-sectional studies that rely on the participants’ self-view measured with self-report questionnaires, the distinguishing role of shame in BPD has less consistently been found in the still small number of studies using ESM during everyday life and experimental paradigms. Since shame has increasingly become a treatment focus in BPD in recent years (e.g., 41), further studies are required to investigate the exact role of shame in the psychopathology of BPD.

In the current study, we aim to contribute to the understanding of shame in BPD by investigating whether individuals with BPD differ from healthy control persons in levels of state shame and its relationship to shame proneness. From our perspective, an experimental investigation of state shame requires a situation during which the participants experience a strong reference to his/her own self in contrast to the use of scenarios during which participants have to take the perspective of another individual and might thereby rely on social cognitive processes such as empathy or imagination abilities of the participants. In our study, we followed the definition of shame as a self-conscious emotion and used the confrontation with one’s own face to activate self-awareness and stimulate self-reflection and self-evaluation by answering questions about the preferences for one’s own face. For this purpose, we measured baseline levels of shame and the change of shame induced by the experimental paradigm. In order to control whether effects are shame specific or only one facet of the overall negative affect characterizing BPD, we additionally assessed several other negative emotions comprising basic emotions such as anger, sadness, disgust, and anxiety as well as self-conscious emotions such as guilt and envy. We examined (i) whether the confrontation with one’s own face is associated with elevated negative emotional responses in BPD, (ii) whether this effect is stronger than in healthy individuals, and (iii) whether this effect is shame-specific. We hypothesized that individuals with BPD respond more intensely with negative emotions when being confronted with one’s own face compared to another one’s face than HCs. We expected that individuals with BPD report higher levels of shame proneness and state shame compared to HCs and that their state shame levels are especially pronounced when being confronted with one’s own self. Additionally, we asked participants to evaluate the pleasantness of the faces presented during the experimental task. We hypothesized that individuals with BPD rate their own face as less pleasant compared to the faces of others and in comparison to HCs. Finally, we investigated to what extent self-reported shame proneness is associated with state shame. We expected a positive correlation of levels of shame proneness with state shame at baseline as well as with the shame response when evaluating the own face during the experimental task.

## 2. Materials and methods

### 2.1. Participants

We recruited a sample of individuals with a BPD and sample of HCs. Participants of the BPD sample were recruited though flyers and verbal advertisement. HCs were recruited from the database of the central project of the KFO 256, a Clinical Research Unit funded by the German Research Foundation dedicated to investigating mechanisms of disturbed emotion processing in BPD (42), the department research website, social networks, and study flyers that were distributed at universities and vocational schools. General inclusion criteria for study participation were an age of 18–25 years and female sex. Inclusion criterion for the BPD sample was the presence of a primary diagnosis of BPD according to DSM-IV (43). Exclusion criteria were the presence of a diagnosis from the schizophrenic disorder spectrum, the presence of acute manic episode or substance dependence. Exclusion criteria for the HC sample were the presence of a mental disorder or current psychotherapeutic/psychiatric treatment. One hundred and nine individuals participated in the study, with 62 being residential or outpatients from the Clinic for Psychosomatic Medicine and Psychotherapy at the Central Institute of Mental Health (CIMH), Mannheim, with a primary diagnosis of BPD and 47 being healthy controls persons.

Diagnoses in the BPD samples have been established by the Structured Clinical Interview for DSM-IV Axis-I Disorders Clinician Version (SCID-CV; 44, 45) and the Structured Clinical Interview for DSM-IV Axis II Personality Disorders (SCID II; 46, 47). HCs were screened for the presence of a current mental disorder using the German version of the Mini International Neuropsychiatric Interview (MINI; 48). All individuals provided written informed consent before participating in the study. The study was approved by the Research Ethics Board II of the Medical Faculty Mannheim of Heidelberg University.

We characterized the samples by assessing sociodemographic features and psychopathology. We measured BPD symptom severity with the short version of the Borderline Symptom List (BSL-23; 49) and severity of syndromes of somatization, depression, and anxiety with the German version of the Brief Symptom Inventory (BSI-18; 50). Additionally, we measured trait self-esteem with the Rosenberg Self Esteem Scale (RSES; 51, 52). In the BSL-23, participants evaluated the severity of BPD symptoms during the previous week in 23 items [5-point Likert scale from 0 (‘*not at all*’) to 4 (‘*very strong*’)]. The BSL-23 mean score ranges from 0 to 4 with higher scores indicating a higher level of BPD symptoms. In the present study, internal consistency was α = 0.96 in the HC sample and 0.95 in the BPD sample. The total score of the BSI-18 is an indicator of general psychological distress (Global Severity Index, GSI) ranging from 0 to 72. Additionally, subscales of somatization, depression, and anxiety are assessed with six items rated on a 5-point Likert Scale ranging from 0 (‘*not at all*’) to 4 (‘*extremely*’). In the present study, internal consistencies were heterogeneous ranging from low to acceptable for the different subscales (α = 0.73 for Somatization, α = 0.55 for Depression, α = 0.68 for Anxiety, and GSI α = 0.87 in the HC sample and α = 0.81 for Somatization, α = 0.76 for Depression, α = 0.78 for Anxiety, and GSI α = 0.91 in the BPD sample). The RSES is a self-report measure of global self-esteem, consisting of 10 items rated on a 4-point Likert Scale from 1 (‘*strongly disagree*’) to 4 (‘*strongly agree*’) that are added up to a total score. In our study, Cronbach’s α was 0.83.

To measure proneness to shame, we used the subscale ‘proneness to shame’ (TOSCA-SHAME) of the short version of the Test of Self-Conscious Affect-3 (TOSCA-3; 32, 53). The short version comprises 11 scenarios describing negative social events (e.g., “While playing around, you throw a ball and it hits your friend in the face”). For each scenario, there are four different statements with possible reactions to the event and participants had to judge how strongly these statements would fit their own behavior on a 5-point Likert scale from 1 (‘*not likely*’) to 5 (‘*very likely*’). The statements correspond to a shame-reaction (e.g., “You would feel inadequate that you cannot even throw a ball”), a guilt-reaction (e.g., “You would apologize and make sure your friend feels better”), an externalization-reaction (e.g., “You would think maybe your friend needs more practice at catching”), and a detachment-reaction (e.g., “You would think: ‘It was just an accident’”). Based on these four statements, we calculated sum scores (range 11–55) for each of the subscales proneness to shame, proneness to guilt, externalization (of blame), and detachment/unconcern. In our study, Cronbach’s α was 0.88 for the shame-proneness scale.

### 2.2. General procedure

The study was conducted in Germany and consisted of two parts: the completion of an online questionnaire, and the experimental paradigm. The online survey comprised questionnaires on sociodemographic data, current severity of psychopathological symptoms, and shame proneness and was created using “Unipark.” Participants received the link to the questionnaire the day before they participated in the experimental paradigm. The experiment was conducted in a laboratory at the Institute for Psychiatric and Psychosomatic Psychotherapy of the Central Institute of Mental Health (CIMH) in Mannheim. At the end of the study, subjects were debriefed, thanked, and they received a small fee for their participation.

Please note that we additionally measured the physiological response in ECG and blushing, but the results will not be presented in this manuscript.

### 2.3. Experimental paradigm and stimulus material

#### 2.3.1. Overview

**Figure 1:**
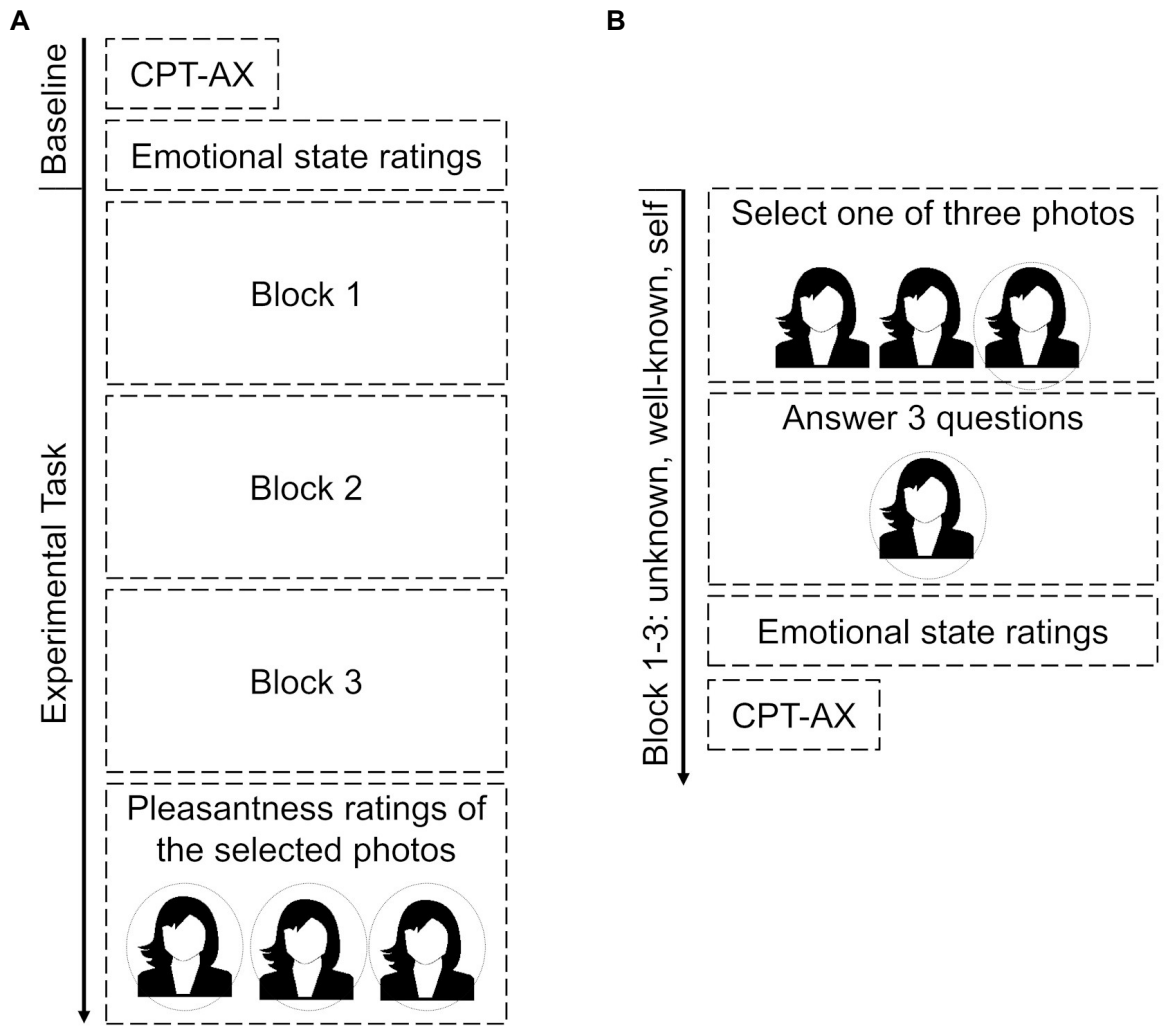
Schematic illustration of the experimental paradigm. Schematic illustration overview of the overall sequence of the experimental paradigm **(A)** and the sequence within a block **(B)**. Please note that the order of the three blocks (unknown, well-known, self) was counterbalanced across participants. CPT-AX, continuous performance task-AX.

An overview of the experimental paradigm is displayed in Figure 1A. The paradigm comprised three blocks during which participants selected one of three images either of themselves (self), of a well-known other person (other well-known) or of a stranger (other unknown) (see Figure 1B). Subsequently, they answered standardized questions about their decisions. At the beginning of the experiment (baseline) and following their answers in each block, participants evaluated their emotional state. Between blocks, participants solved a cognitive task to reduce carry-over effects. The order of the three blocks was counterbalanced across participants. At the end of the experiment, participants rated for each of the three portraits they liked best how pleasant or unpleasant they found the respective face.

Participants were informed that their faces would be filmed and their answers would be audiotaped during the entire experiment. Participants were seated at a table with a computer and a video camera in frontal orientation to them. The experimental task was programmed in Presentation^®^ (nbs.neurobs.com).

#### 2.3.2. Stimulus material

During the experimental task, we manipulated the self-reference of the facial stimuli by presenting in the three blocks either a photo of the participant herself (self), a well-known (other-well-known), or an unknown (other-unknown) person. The photos of the participants were taken before the start of the experiment, i.e., three standardized frontal portrait photos (upright) of the participants were taken in a simulated photo studio with standardized lighting. As stimuli in the other-well-known condition, three photos of Emma Watson as a well-known person were used. We selected Emma Watson for the other-well-known condition based on a previously conducted survey to ensure that the person is known to all persons with the highest possible probability and has a comparable age to the participants. As stimulus in the other-unknown condition, three photos of an unknown female person were used to control effects of familiarity (e.g., 54, 55). These photos were taken under the same situational conditions as the photos of the participants.

#### 2.3.3. Experimental task blocks

Each block of the experimental task comprised four parts.

Part A. At the beginning of each block, participants were presented—depending on the experimental condition—with either the three previously taken photos of their own face, three photos of Emma Watson, or three photos of an unknown person presented on a computer screen in front of the participants. Participants were instructed to select one of these three photos according to their best liking [instruction: “You will now see the three photos of (yourself/a person you are well-known with/an unknown person). Take some time and decide which of these three photos you like best.”]. Participants signaled their decision by moving a cursor with the computer mouse to one of the facial stimuli and pressing a mouse button.

Part B. Following the selection of an image and a break of 60 s, the selected face was shown on the computer screen and participants had to answer different questions about the reasons for their decision (questions: “I will now ask you some questions about the photo. Please speak your answer loud and clear into the camera: Why did you choose this photo as the most beautiful? Which aspect of the face do you like best? Why do you like this particular aspect best?”). For each question, participants had 90 s to respond. They signaled the end of their answer by pressing a button. During the questions and the participants’ answers, the chosen photo remained on the screen. Instructions and questions were delivered by a prerecorded audio file *via* headphones. The audio instruction was chosen to prevent experiential avoidance of viewing the presented faces, to reduce possible interferences between presentations of the visual stimuli, and to create the feeling of a social context situation.

Part C. After answering the different questions, the presentation of the facial stimulus was finished and participants rated how intensely they were experiencing different emotions. Additionally to shame, participants assessed the intensity of the self-conscious emotions guilt and envy as well as the basic emotions anger, disgust, sadness, and anxiety to differentiate whether the experimental manipulation affected specifically the experience of shame or negative affect in general. Negative emotions were presented intermixed with positive emotions used as distractors in order to reduce the priming of a negative evaluation bias (pride, interest, joy, satisfaction). All emotions were rated on a visual analog scale [ranging from 0 (not at all) to 100 (very much)] presented on the computer screen by moving a cursor with a computer mouse on the scale and confirming their rating by pressing a mouse button. Please note that the same visual analog scale was used for the ratings of pleasantness of the presented faces at the end of the experiment.

Part D. Each block was terminated by a cognitive task (65 trials, duration 2.5 min) to reduce carry over effects. We chose a cognitive continuous performance task during which the participants had to press a particular button when the target letter X was presented and preceded by the letter A (56).

### 2.4. Statistical analysis

We compared the two groups with independent t-tests for age and questionnaire data and with the Cochrane-Armitage test for education. As dependent variables in the experimental task, we analyzed (a) mean intensity ratings of the emotional state averaged for each participant across the negative emotion categories and (b) intensity ratings of the single negative emotion categories. We compared the mean intensity ratings between groups during baseline with an independent *t*-test and during the experimental task with a 2 × 3 mixed ANOVA design with ‘group’ as between-subject factor (BPD, HC) and ‘reference’ as within-subject factor (unknown, well-known, self). Assumptions of normality, sphericity (Kolmogorov–Smirnov tests, visual inspection of graphic plots, Mauchly’s), and equality of variances (Levene’s test) were checked. Kolmogorov–Smirnov test indicated a violation of the normal distribution assumption of the residuals. However, as the visual inspection of the graphic plots indicated an approximate assumption of normal distribution, parametric tests for these analyses were conducted. To analyze the importance of shame as a distinct emotion category, we extended the analyses with ‘emotion category’ as an additional within-subject factor. Since the intensity ratings for each emotion category are ordinal data, we used a rank-aligned nonparametric ANOVA (57) for analyses. For baseline ratings, we applied a 2 × 7 mixed ANOVA with the between-subject factor ‘group’ (HC, BPD) and the within-subject factor ‘emotion category’ (shame, guilt, envy, anger, disgust, sadness, and anxiety). To reduce the complexity of the design for the experimental task, we combined the two other-referential conditions (unknown, well-known) by averaging both conditions resulting in a 2 × 2 × 7 mixed ANOVA with the factors ‘group’, ‘reference’ (other, self) and ‘emotion category’. Please note that we used baseline-corrected rating scores in the analysis of the experimental task to control for difference in intensity ratings between groups that existed independently of the experimental task already during baseline ratings. Finally, we analyzed pleasantness ratings of the facial stimulus presented in the unknown, well-known and self-referential condition during the experimental task by means of a 2 × 3 rank-aligned nonparametric mixed ANOVA. We corrected degrees of freedom according to Greenhouse–Geisser. As *post-hoc* analyses, we conducted Mann–Whitney U Test and nonparametric ANOVA sub-designs, respectively. To control for multiple testing, we applied a Bonferroni correction for the families of pairwise comparisons. Significance levels corrected for multiple testing are marked as p_Bonferroni_. We report effect sizes according to the applied statistical approach.

Correlational analyses of shame-proneness with state shame at baseline and experimentally induced state shame were conducted using Spearman’s correlation coefficient. Significance level for all analyses was *α* = 0.05, two-tailed. Data analysis was conducted with IBM SPSS Statistics Version 23 and matlab R2022a.

## 3. Results

### 3.1. Sample description

The BPD and HC groups were balanced for age (*t* = 1.60, *p* = 0.113) and education (*Χ^2^* = 1.51, *p* = 0.680). Mean age of the BPD sample was 20.84 ± 2.09 years (range 18–25 years) and 21.49 ± 2.13 years (range 18–25 years) in the HC sample. The BPD group reported higher levels of BPD symptoms (BSL-23), a higher general psychological distress (BSI-18), and a lower self-esteem compared to HCs (all *ps* < 0.05). Further details are depicted in Table 1.

**Table 1:** Sample characteristics.

	BPD (*N* = 62)	HC (*N* = 47)	Test statistic	*p*
**Demographics**				
Age^a^	20.84 (2.09)	21.49 (2.13)	1.60	0.113
Education, *n* (%)^b^			1.51	0.680
Low (primary school, lower vocational education)	7 (11.3)	3 (6.4)		
Intermediate (secondary school, vocational education)	29 (46.8)	24 (51.1)		
High (higher vocational education, university)	25 (40.3)	18 (38.3)		
University degree	1 (1.6)	2 (4.3)		
**Current comorbidities, *n* (%)**				
Affective disorder	49 (79.0)	0		
Anxiety disorder	17 (27.4)	0		
Posttraumatic stress disorder	21 (33.9)	0		
Substance abuse	5 (4.8)	0		
Eating disorder	14 (22.6)	0		
Obsessive compulsive disorder	2 (3.2)	0		
Other disorder	8 (12.9)	0		
Current treatment, *n* (%)	62 (100)	0		
Residential patients	43 (69.4)	0		
Outpatient	19 (30.6)	0		
Psychopharmacological treatment *n* (%)	57 (91.9)	0		
**Clinical characteristics**				
BSL-23^a^	2.3 (0.9)	0.2 (0.5)	14.5	<0.001
BSI-18 Global severity ^a^	56.8 (15.1)	24.2 (7.1)	13.6	<0.001
Somatization^a^	19.1 (5.6)	7.9 (2.6)	12.7	<0.001
Depression^a^	17.3 (5.5)	7.7 (2.4)	11.2	<0.001
Anxiety^a^	20.4 (5.1)	8.6 (2.7)	14.3	<0.001
Rosenberg Self-Esteem Scale^a^	17.5 (5.3)	34.2 (4.7)	17.1	<0.001
**TOSCA-3**				
Shame^a^	44.1 (6.2)	29.0 (7.4)	11.5	<0.001
Guilt^a^	47.7 (6.8)	45.6 (4.5)	1.9	0.060
Externalization of blame^a^	21.2 (5.6)	22.0 (5.7)	−0.7	0.469
Detachment/Unconcern^a^	22.7 (6.6)	30.6 (5.2)	−6.8	<0.001

BPD, borderline personality disorder; HC, healthy control; BSL, borderline symptom list; BSI-18, brief symptom inventory-18; TOSCA-3, test of self-conscious affect scale-3. ^a^*T*-test; ^b^Cochran-Armitage test.

### 3.2. Self-reported proneness to shame

Individuals with BPD reported a higher proneness to shame in the TOSCA-subscale (TOSCA-SHAME) compared to HCs (*t* = 11.51, *p* < 0.001, *d* = 2.24). Please note that the individuals with BPD reported also a lower score in the TOSCA subscale “detachment and unconcern” compared with HCs (*t* = 6.76, *p* < 0.001, *d* = −1.31), but did not differ significantly from HCs in the TOSCA subscales “proneness to guilt” and “externalization of blame” (*ps* > 0.05).

### 3.3. Experimental task

#### 3.3.1. Baseline

**Figure 2:**
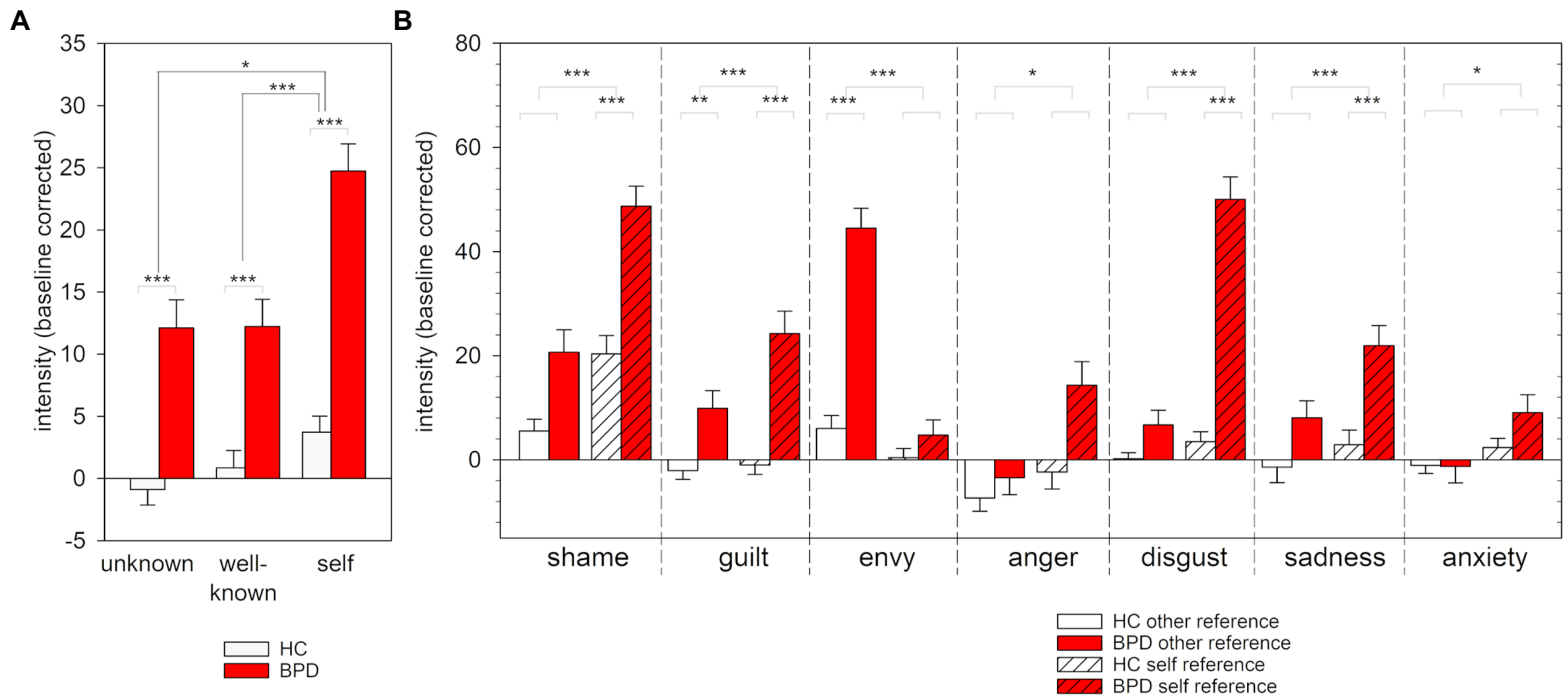
Ratings of the intensity of negative emotions for BPD and HC at baseline. Mean and standard error of ratings for negative emotions at baseline. **(A)** Mean intensity rating averaged across the different negative emotions. **(B)** Intensity ratings for the different negative emotions. ^*^p < 0.05, ^**^p < 0.01, ^***^p < 0.001 (Bonferroni-corrected).

At baseline, individuals with BPD experienced higher intensity of negative emotions compared with HCs (*t* = 6.52, *p* < 0.001, *d* = 1.14, Figure 2A).

Analyses of the different emotion categories between groups in a 2 × 7 rank-aligned non-parametric ANOVA revealed that differences in intensities between groups varied between the emotion categories (interaction effect “group * emotion”: *F*(6, 620) = 14.83, *p* < 0.001 *Cohen’s f* = 0.33; Figure 2B). *Post hoc* comparisons between groups revealed higher intensities for shame, guilt, anxiety, sadness, and anger in the BPD compared to the HC group (all *p_Bonferroni_s* < 0.05,). In contrast, both groups did not differ significantly on baseline levels of envy (*p* = 0.163) and only at a trend level on baseline levels of disgust (*p_Bonferroni_s* < 0.10). For further details, see Supplementary Material Table S1.

#### 3.3.2. Changes of the emotional state during task solving

**Figure 3:**
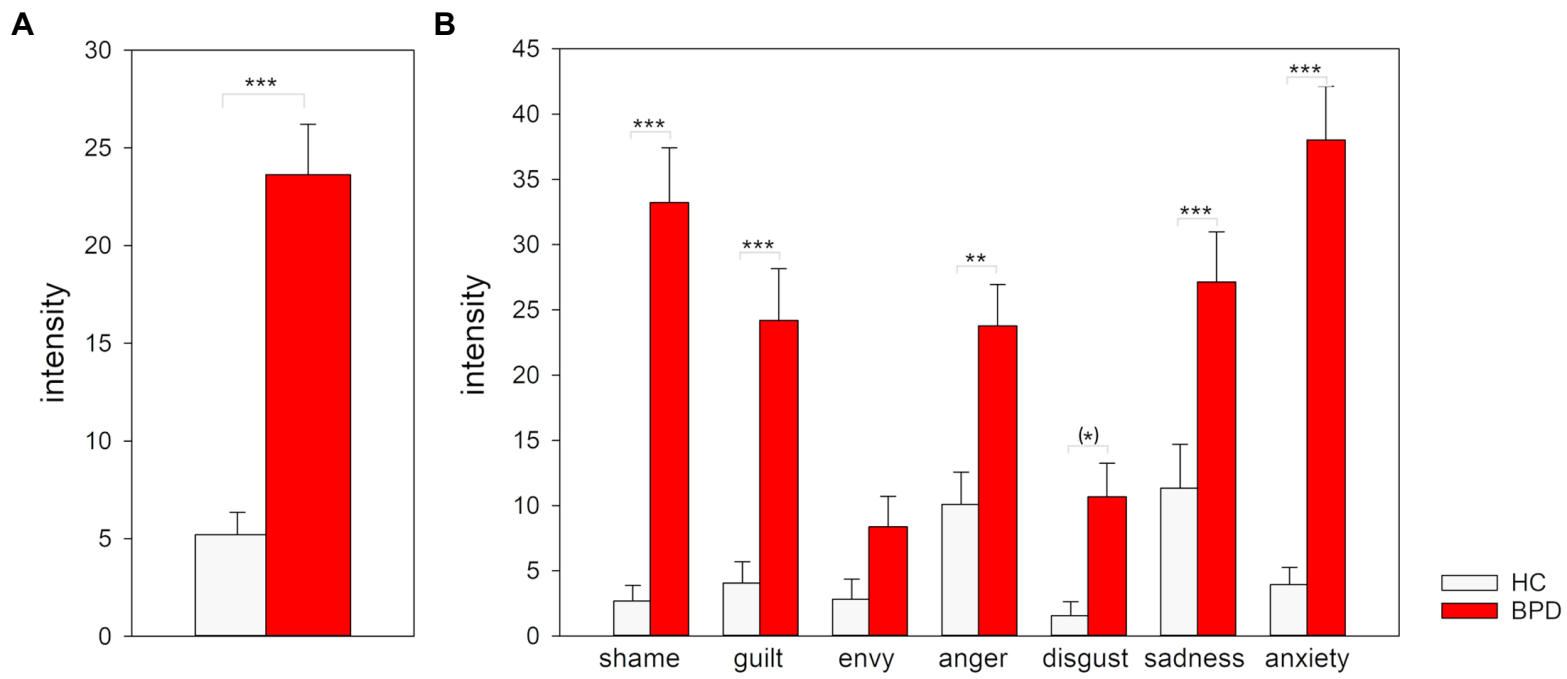
Ratings of the intensity of negative emotions for BPD and HC during the experimental task. **(A)** Mean and standard error for baseline-corrected intensity ratings averaged across the different negative emotions in the three experimental task conditions unknown, well-known, and self. **(B)** Mean and standard error for baseline-corrected intensity ratings for the different negative emotion categories in the other- and self-referential condition. Ratings of the unknown and well-known condition were combined in the other-referential condition. Scores >0 indicate an increase in the intensity ratings during the experimental task compared to baseline. ^*^p < 0.05, ^**^p < 0.01, ^***^p < 0.001 (Bonferroni-corrected).

Individuals with BPD reported higher levels in the intensity of negative emotions during the experimental task in relation to the baseline than the HC group across all experimental conditions as indicated by the higher baseline-corrected rating scores (main effect “group” *F*(1, 107) = 39.65, *p* < 0.001; *Cohen’s f* = 0.62, Figure 3A). This difference between groups was influenced by the experimental condition (interaction “group * condition” *F*(2,214) = 9.05, *p* < 0.001; *Cohen’s f* = 0.30). In *post-hoc* analyses, we compared pairs of the three experimental conditions in ANOVA sub-designs. These analyses revealed differences between groups particularly for the comparison of the self-referential condition with both other-referential conditions: Negative emotions were more intense in the BPD group than in the HC group when the own face was presented compared to both an unknown and a well-known face (interaction effects ‘group * condition’ in sub-design ‘unknown/self’ *F*(1, 107) = 8.47, *p* = 0.004, *p_Bonferroni_* = 0.012, *Cohen’s f* = 0.28; ‘well-known/self’: *F*(1, 107) = 16.65, *p* < 0.001, *p_Bonferroni_* < 0.001, *Cohen’s f* = 0.40; ‘unknown/well-known’: *F*(1, 107) = 0.59, *p* = 0.446, *p_Bonferroni_* = 1.00, *Cohen’s f* = 0.07).

To analyze whether shame plays a central role when particularly one’s own face in comparison to another face is presented, we compared the self-and other-referential task condition depending on the emotion categories between groups. Since there were no differences between ratings for unknown and well-known faces, we combined these two experimental conditions to one ‘other-referential’ condition (for further details see Supplementary material Figure S1).

Results of the 2 × 2 × 7 nonparametric rank-aligned ANOVA revealed that the emotion rated and the face evaluated influenced differences between groups (interaction effect ‘group * reference * emotion’: *F*(6, 642) = 52.75, *p* < 0.001, *Cohen’s f* = 0.70; Supplementary Material Table S2). In *post-hoc* analyses, we compared the groups in ANOVA sub-designs separately for the different emotion categories (Table 2; Figure 3B). These analyses revealed that individuals with BPD reported stronger differences between the other-referential and self-referential condition for all emotions compared to HC (all interaction effects *p* < 0.05). Effect sizes were large for disgust (*Cohen’s f* = 0.84) and envy (*Cohen’s f* = 0.75), small for anxiety (*Cohen’s f* = 0.29), and medium for the other emotions (*Cohen’s f* = 0.30 to *Cohen’s f* = 0.75). BPD patients reported more intense negative emotions in the self-referential condition compared with the other-referential condition than HC, except for envy for which the level was higher in the other-referential condition compared to the self-referential condition. In consequence, BPD patients reported higher levels of shame, guilt, disgust and sadness than HC when confronted with the own face, as well as a higher level of envy when confronted with another one’s face. Please note that the interpretability of the main effects group and reference is restricted for most emotions by the higher-order interaction effect (for further details see Supplementary Material Tables S3, S4).

**Table 2:** Results of 2 × 2 rank-aligned ANOVA sub-designs for the different negative emotion categories.

	Main effect group			Main effect reference			Interaction effect group x reference		
	*F*(1,107)	*p*	Cohen’s *f*	*F*(1,107)	*p*	Cohen’s *f*	*F*(1,107)	*p*	Cohen’s *f*
Shame	22.92	**<0.001**	0.46	92.15	**<0.001**	0.93	10.70	**0.001**	0.32
Guilt	27.63	**<0.001**	0.51	32.04	**<0.001**	0.55	26.48	**<0.001**	0.50
Envy	59.61	**<0.001**	0.75	115.83	**<0.001**	1.04	60.05	**<0.001**	0.75
Anger	2.91	0.091	0.16	27.13	**<0.001**	0.50	9.68	**0.002**	0.30
Disgust	72.42	**<0.001**	0.82	117.93	**<0.001**	1.05	75.78	**<0.001**	0.84
Sadness	17.75	**<0.001**	0.41	41.27	**<0.001**	0.62	16.31	**<0.001**	0.39
Anxiety	1.63	0.205	0.12	22.35	**<0.001**	0.46	9.22	**0.003**	0.29

Significance levels below the Bonferroni corrected threshold of *p* < 0.007 for *α* = 5% are marked in bold.

### 3.4. Pleasantness of the faces

**Figure 4:**
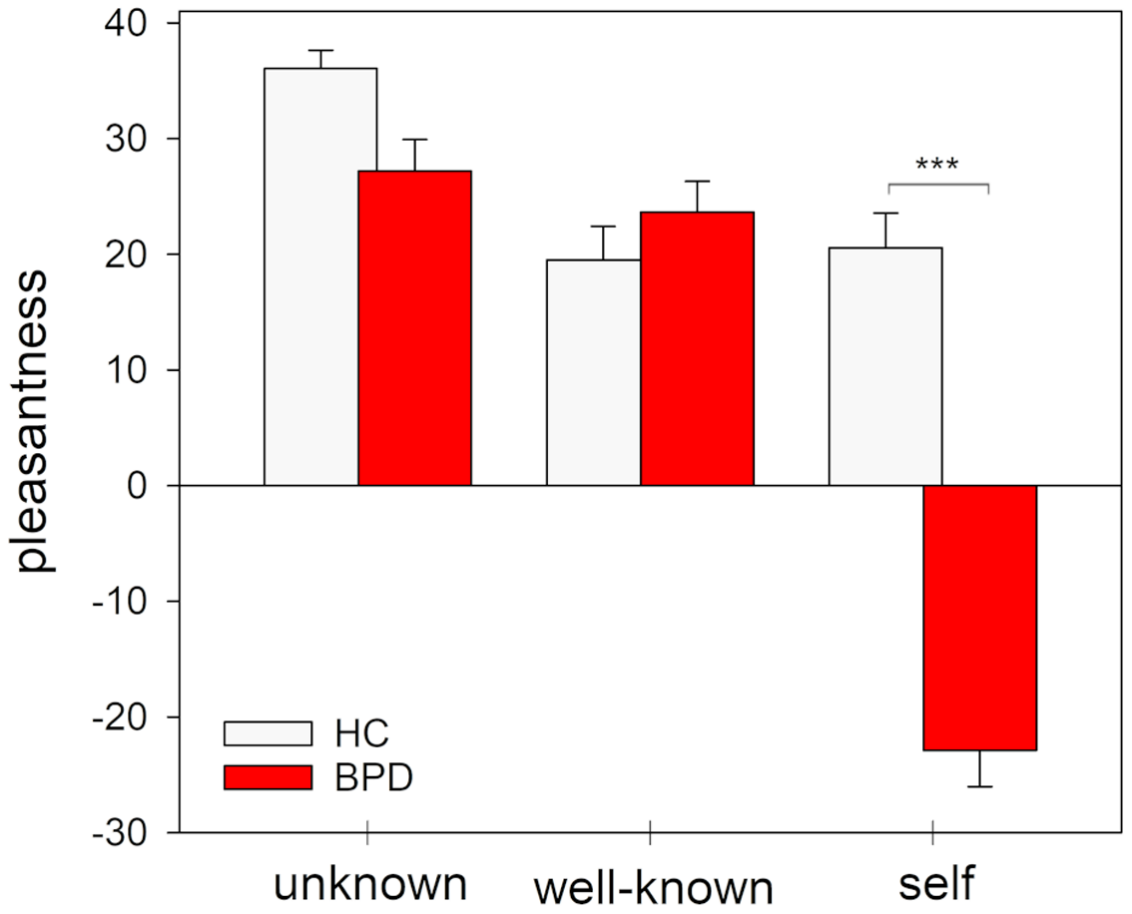
Pleasantness ratings of the images of the facial stimuli presented during the unknown, well-known and self-referential condition of the experimental task. Mean and standard error for pleasantness ratings. ^***^p < 0.001 (Bonferroni-corrected).

Results of the nonparametric 2 × 3 rank aligned ANOVA revealed that individuals with BPD differed from HCs in judging the pleasantness of the presented faces in dependence of the presented faces (interaction ‘group x condition’: *F*(2, 238) = 42.52, *p* < 0.001; *Cohen’s f =* 0.56). *Post-hoc* tests revealed that both groups differed only in ratings of pleasantness when judging the own face (*Z* = −7.10, *p_Bonferroni_* < 0.001, *r* = −0.68): BPD patients rated their own faces markedly as less pleasant than the HC group. In contrast, both groups did not differ in judgments of pleasantness for unknown and well-known faces (for well-known faces: *Z* = −1.23 *p_Bonferroni_* = 0.220, *r* = −0.12; for unknown faces: *Z* = −1.58, *p_Bonferroni_* < 0.114, *r* = −0.15). Results are depicted in Figure 4.

### 3.5. Relationship between state shame and shame proneness

Correlational analyses across all participants revealed that participants with higher shame proneness as assessed with TOSCA-SHAME showed higher levels of baseline shame before the experimental task (*r* = 0.43, *p* < 0.001), higher levels of state shame ratings during the confrontation with their own face (*r* = 0.35, *p* < 0.001) as well as a higher level of shame in the self-referential compared to the other-referential experimental condition (*r* = 0.23, *p* < 0.017). However, when analyzing these correlations separately for both groups, there were no significant correlations between these trait and state measures neither in the HC nor in the BPD group (all *rs* ≤ ±0.04, all *ps* ≥ 0.763; see Supplementary material Figure S2).

## 4. Discussion

In the present study, we investigated negative emotional responses with a specific focus on shame in individuals with BPD compared to HCs during the experimental confrontation with one’s own face. In addition, we examined whether shame proneness is related to levels of state shame. Our findings revealed higher levels of negative emotions except for disgust and envy in BPD compared to HCs at baseline. During the experimental paradigm, individuals with BPD reported higher levels of negative emotions than HCs, with differences between the two groups being largest for the own-face in comparison to the unknown or the well-known face condition. However, different emotions were differentially affected by the self in comparison to the other referential evaluations: Compared to HCs, individuals with BPD reported higher scores particularly for disgust when seeing one’s own picture which is in line with their negative self-image. Moreover, seeing others or celebrities triggered a high degree of envy in BPD patients when compared to HCs which might similarly reflect the patients’ negative self-concept triggered in social comparison situations. Moreover, confrontation with one’s own face resulted in higher levels of various negative emotions including shame, guilt, and sadness although with smaller effect sizes as those observed for disgust. In addition, individuals with BPD rated their own face as more unpleasant than an unknown or well-known face compared with HCs. While the BPD group showed a higher shame proneness than the HC group, a correlation of higher shame proneness as assessed with TOSCA-SHAME was related to higher levels of state shame at baseline and during the experimental task across all participants, but not within the single groups.

With regard to state shame assessed as baseline of the experimental task, our findings are in line with previous studies suggesting elevated levels of negative affect in individuals with BPD compared to HCs. Several studies have already shown alterations in the processing of negative self-conscious emotions such as shame, self-disgust, or self-contempt central to BPD psychopathology (8–10). However, in contrast to our study, most of these previous studies have assessed rather proneness to a specific emotion than state emotional responses and have used scenario-based questionnaires in which the respondents take the perspective of a protagonist without any direct self-reference.

Regarding emotional state ratings during the experimental task, our findings suggest differences in emotional reactivity in BPD compared to HCs depending on the experimental condition and varying between negative emotions. This is in contrast to the results of Scheel et al. (40), who did not find any group differences between specific emotional states after experimental shame induction. A more detailed analysis of our results showed that this effect resulted particularly strong from differences between groups in disgust and envy: BPD patients reported higher levels of disgust when confronted with their own face, as well as higher levels of envy when confronted with the face of an unknown or well-known other individual compared with HC. Furthermore, with regard to the specificity of single emotions, our results are in accordance with those of Gratz et al. (36), suggesting that differences between negative emotional responses in BPD compared to healthy individuals depend on contextual cues and specific triggers, in our study being confronted with the own face in contrast to the face of another person, and vary between different negative emotions. However, higher levels of negative emotions triggered by the confrontation with one’s own face were also observed for shame, guilt, anger, sadness, and anxiety in BPD. This finding might indicate a more complex emotional response in the sense of activating emotional networks instead of individual emotions in BPD. The finding of elevated state levels of disgust in BPD is in line with the current state of research: Previous studies have shown an increased tendency to disgust proneness (11, 58–60) and state disgust in BPD (60). Especially, higher levels of self-disgust have shown to be related to more pronounced severity of BPD psychopathology (61). Furthermore, previous findings suggest that self-disgust is related to Non-Suicidal-Self-Injuries (NSSI) in BPD and beyond (58, 61–64). Since self-disgust is often considered a facet of self-criticism, our results also fit against the background of increased self-criticism in BPD compared to a general population sample as well as other clinical samples (65).

In addition, recent emotion theories assume both maladaptive and adaptive facets of shame: They emphasize that shame can also serve socially regulatory and protective functions important for the development and maintenance of interpersonal relationships (66, 67). In consequence, one might consider the extent to which automatic, fast, and pre-attentive development of disgust might represent a maladaptive shame response in BPD that leads to self-damaging behavior (58, 61, 62) rather than socially adaptive action tendencies (e.g., appeasing a social group). This is in line with previous research suggesting a reduced capability to control disgust responses in individuals with BPD compared to healthy controls (60). Although the confrontation with one’s own face was not accompanied by exclusively higher levels of shame, our findings of elevated levels of baseline shame and state shame during the confrontation with the own face indicate a specific importance of this emotion in BPD which is in line with previous results on elevated shame proneness (11, 67) and state shame (34–36). In the context of shame as self-conscious emotions, it is also assumed that these emotions do not only arise from self-referential processes including self-awareness, self-reflection, and self-evaluation but also affect socio-cognitive processes in social interactions (9): Shame has been linked to increased self-awareness and tendencies to avoid social interactions (68–70), whereas, for example, the self-conscious emotion of guilt is assumed to increase empathy and cooperative behaviors, decreases self-focused attention while directing attention toward social interaction partners (68, 70, 71). In case of BPD, our findings revealed both higher levels of shame and guilt. Based on the assumption that both emotions are associated to different behaviors, one might speculate whether our findings point to the participants’ problems in differentiating both emotions or a mechanism contributing through conflicting behavioral consequences to the affective and social instability characterizing BPD.

When interpreting our results in the context of previous research, it seems also important to discuss the influence of different experimental shame inductions on emotional responses: Previous studies have used social context as a trigger for shame [e.g., negative feedback in the study of Gratz et al. (36) or a failed job interview in the study of Scheel et al. (40)]. In contrast, we used the exposure to the own face without an explicitly given social context shifting the emphasis from violation of social norms in the view of others to violation of one’s own norm. However, the fact that the participants did not only look at their own face, but also described the merits of it and justified their decision in our study, apparently led to higher levels of both disgust and shame.

Interestingly, in our study individuals with BPD reported significantly higher intensity of envy when being confronted with another face in contrast to the own face compared to HCs. To our knowledge, there is no previous study focusing on the specific emotion of envy in BPD patients. Given the high importance of this emotion in social comparisons (72), future studies are needed to clarify to what extent the BPD criteria of instability in self-image and relationships are associated with elevated levels of envy.

Furthermore, our results reveal that individuals with BPD evaluated particularly their own face as less pleasant compared to other referential faces than HCs. This is in line with previous studies, suggesting that BPD is characterized by negative self-evaluations including a negative self-concept with low levels of self-esteem, a tendency to avoid self-awareness cues, and higher levels of negative self-conscious emotions (9, 73).

To our knowledge, this study was the first to investigate the relationship between shame proneness and negative emotional state responses with a focus on shame. Although our findings suggest a positive correlation between shame proneness and levels of state shame during the confrontation with one’s own face across all participants, this correlation could not be found in the two individual samples. Although it can be debated to what extent the relation seen across all participants might simply reflect the group difference in the investigated variables, we used this approach to examine the relationship between shame proneness and state shame across a broader range of evaluations. Nevertheless, further studies with larger sample sizes for both the BPD and HC group with a higher variability of ratings within each group are needed to further investigate the interplay between trait and state measures. However, an alternative explanation for the lack in the association between trait and state measures might be that both differed in their relation to the self as well as in the presence of a social context: The TOSCA SHAME captures shame proneness on the basis of predetermined social scenarios in which the respondent has to put himself into the protagonist’s perspective. In contrast, our study emphasized the relation to the self through the confrontation with one’s own face without the need to ‘step in another one’s shoes. Thus, both approaches can be assumed to capture different processes. This interpretation is supported by exploratory analyses of the associations between our shame measures and the severity of BPD psychopathology: When exploring these relationships, we found that a higher BSL-23 score was related to a higher shame proneness in both groups (HC: *r_s_* = 0.49, *p* < 0.001; BPD: *r_s_* = 0.53, *p* < 0.001), but not to the shame ratings of the experimental task (all *ps* > 0.200). Moreover, our paradigm did not involve a direct social interaction. However, one might speculate whether answering questions about the merits of one’s own face with the awareness of being video- and audiotaped constitutes an “indirect” social situation. Whether a stronger shame response would be evoked in the actual presence of others has to be investigated in future studies. Beyond these differences, the state shame response might depend on maladaptive automated processes such as state self-disgust thereby inducing emotions of a different quality as the measures of shame proneness. This has several implications for the psychotherapeutic treatment of impairments in BPD: on the one hand, it implies the change of automatically activated (maladaptive) emotional responses and action tendencies (e.g., opposite action in DBT) and, on the other hand, the change of a rather persistent tendency to feel negative self-conscious emotions such as shame in a variety of situations. For example, Compassion Focused Therapy (CFT) according to Gilbert (74) offers an approach that has already proven to be effective in reducing proneness to shame and self-criticism in other clinical samples. Since our results suggest a more complex emotional event, it would be important to take the multiple emotional changes into account rather than focusing exclusively on the attenuation of a single emotion.

The major strength of our study represents the experimental manipulation of shame as a self-referential construct in a controlled laboratory setting. Nevertheless, some limitations of our study should be kept in mind when interpreting the results: These include the restricted generalizability of our findings, since only women were included in the current study. Since there is evidence of gender differences between men and women in the experience of shame (for meta-analysis see 75), the findings cannot be generalized to males. Moreover, we only included women with age between 18 and 25 in the study. Although this restricts the generalizability of the findings to other age and gender groups, it was necessary in our study to use face photos comparable to the gender and age of all participants for a well known and unknown face while simultaneously presenting the same stimuli in the other-referential conditions across participants. Regarding baseline, it should be taken into account that the participants’ photos were taken before baseline, so that anticipatory effects on the baseline measurement cannot be excluded. However, in contrast to previous studies that specifically evoked anticipatory effects (76), there was no corresponding instruction in our study. Since we reported emotion ratings during the experimental task corrected for baseline, anticipatory effects on the emotional state at baseline might have reduced the response during the experimental conditions. Another limitation concerns the attractiveness of the known (Emma Watson) and unknown person for whom the study did not control. It is possible that the participants rated both persons as significantly more attractive than themselves, which particularly intensified the difference to their own face. However, previous findings on body self-evaluations in BPD suggest that the face is rated least negatively in individuals with BPD compared to other parts of the body (77). This implies that the difference to other people should be the smallest for this area of the body. Nevertheless, further investigations are needed that capture state self-disgust and shame in BPD without reference to the own appearance. In addition, we focused on the investigation of negative self-conscious emotions, whereas positive self-conscious emotions such as pride or self-satisfaction were not taken into account. Based on previous findings of a markedly negative self-image in BPD (9), it can be assumed that the confrontation with one’s own face in BPD is accompanied by low levels and reduced variance in emotions with positive valence compared with HCs. Exploratory analyses of the ratings of positive emotions, which we used as distractors in the current study, support the relevance of positive emotions as one facet of changes in self-referential processing in BPD: Individuals with BPD reported not only lower intensities of positive emotions at baseline compared with HC, but showed also even lower intensities of positive emotions compared with baseline levels when confronted with the own face during the experimental task (for further details see Supplementary material B; Supplementary material Figure S3; Supplementary material Tables S5, S6). Taking recent advances in positive psychology and therapeutic approaches to strengthen resilience into account, further studies are needed to investigate the relevance of self-related positive emotions and their potential for therapeutic interventions. Finally, each of the different negative emotions participants assessed in this study is highly complex regarding their communicative value, their extent of self-reference, their dependency on social comparison processes, and their relevance in BPD. This implies that a composite score across the different categories has to be interpreted with care. Moreover, participants evaluated their emotional state following the confrontation with one’s own face in a specific experimental context, that is, combined with the confrontation with the face of a well-known and unknown person. This might have differentially intensified the influence of social comparison processes on the evaluation of the different emotion categories.

In conclusion, our study is the first experimental study on negative emotional response with a focus on shame and its relationship to shame proneness in BPD in comparison to HCs using the own face as a cue inducing self-awareness, self-reflection, and self-evaluation as important features of self-conscious emotions. Our data confirm previous results of a markedly negative self-image in BPD and high shame proneness. In addition, our results point to the importance of disgust and envy as elevated self-conscious emotions in BPD, which should be further investigated in future research and taken into account as important target emotions in therapeutic interventions.

## Data availability statement

The raw data supporting the conclusions of this article will be made available by the authors, without undue reservation.

## Ethics statement

The studies involving human participants were reviewed and approved by Ethikkommission II der Universität Heidelberg Medizinische Fakultät Mannheim. The patients/participants provided their written informed consent to participate in this study.

## Author contributions

MBi, SL, MBo, and LL contributed to the conception and design of the study. MBi and SL organized the data base and acquisition. MBi and SL performed the data analysis. MBi, MBo, and SL contributed to interpretation of data for the work. MBi wrote the first draft of the manuscript. All authors contributed to the article and approved the submitted version.

## Conflict of interest

The authors declare that the research was conducted in the absence of any commercial or financial relationships that could be construed as a potential conflict of interest.

## Publisher’s note

All claims expressed in this article are solely those of the authors and do not necessarily represent those of their affiliated organizations, or those of the publisher, the editors and the reviewers. Any product that may be evaluated in this article, or claim that may be made by its manufacturer, is not guaranteed or endorsed by the publisher.
