# Supplementary material for: Shame, self-disgust, and envy: An experimental study on negative emotional response in borderline personality disorder during the confrontation with the own face
Source: Front Psychiatry. 2023 Mar 8;14:1082785. doi: 10.3389/fpsyt.2023.1082785 (PMC10030617; doi:10.3389/fpsyt.2023.1082785)
Supplement: Supplementary file 1 [file Data_Sheet_1.docx]

Supplementary Material

Shame, self-disgust and envy: An experimental study on negative emotional response in borderline personality disorder during the confrontation with the own face

Miriam Biermann^1,2*^, Anna Schulze^2^, Ruben Vonderlin^1^, Martin Bohus^1,3,4^, Lisa Lyssenko^5^, & Stefanie Lis^1,2^

^1^Department of Psychiatric and Psychosomatic Medicine, Central Institute of Mental Health, Medical Faculty Mannheim, Heidelberg University, Mannheim, Germany

^2^Department of Clinical Psychology, Central Institute of Mental Health, Medical Faculty Mannheim, Heidelberg University, Mannheim, Germany

^3^Department of Clinical Psychology, Ruhr University Bochum, Bochum, Germany

^4^McLean Hospital, Harvard Medical School, Boston, MA, USA

^5^Department of Public Health, Freiburg University of Education, Freiburg, Germany

*** Correspondence:**Miriam Biermann
Miriam.Biermann@zi-mannheim.de

**Supplementary Material A**

**Table S1.** *Between group comparisons for negative emotional states at baseline*

| Baseline emotion | *Z* | *p*  _uncorrected_ | | *p*  _Bonferroni corrected_ | | effect size *rc* | |  |
| --- | --- | --- | --- | --- | --- | --- | --- | --- |
| Shame | -5.85 | < .001 | < .001 | | 0.65 | |  | |
| Guilt | -3.90 | < .001 | < .001 | | 0.35 | |  | |
| Envy | -1.39 | .163 | 1.000 | | 0.02 | |  | |
| Anger | -3.53 | < .001 | .003 | | 0.25 | |  | |
| Disgust | -3.19 | .001 | < .010 | | 0.29 | |  | |
| Sadness | -3.95 | < .001 | < .001 | | 0.40 | |  | |
| Anxiety | -6.16 | < .001 | < .001 | | 0.63 | |  | |

*Note.* Mann-Whitney-U-Tests between individuals with BPD and HCs for negative emotions at baseline.

*rc* = rank biserial correlation, *p*_Bonferroni corrected_ corresponds to correction for seven pairwise comparisons

**Figure S1.** Ratings of negative emotions for the unknown and well-known condition of the experimental task

**
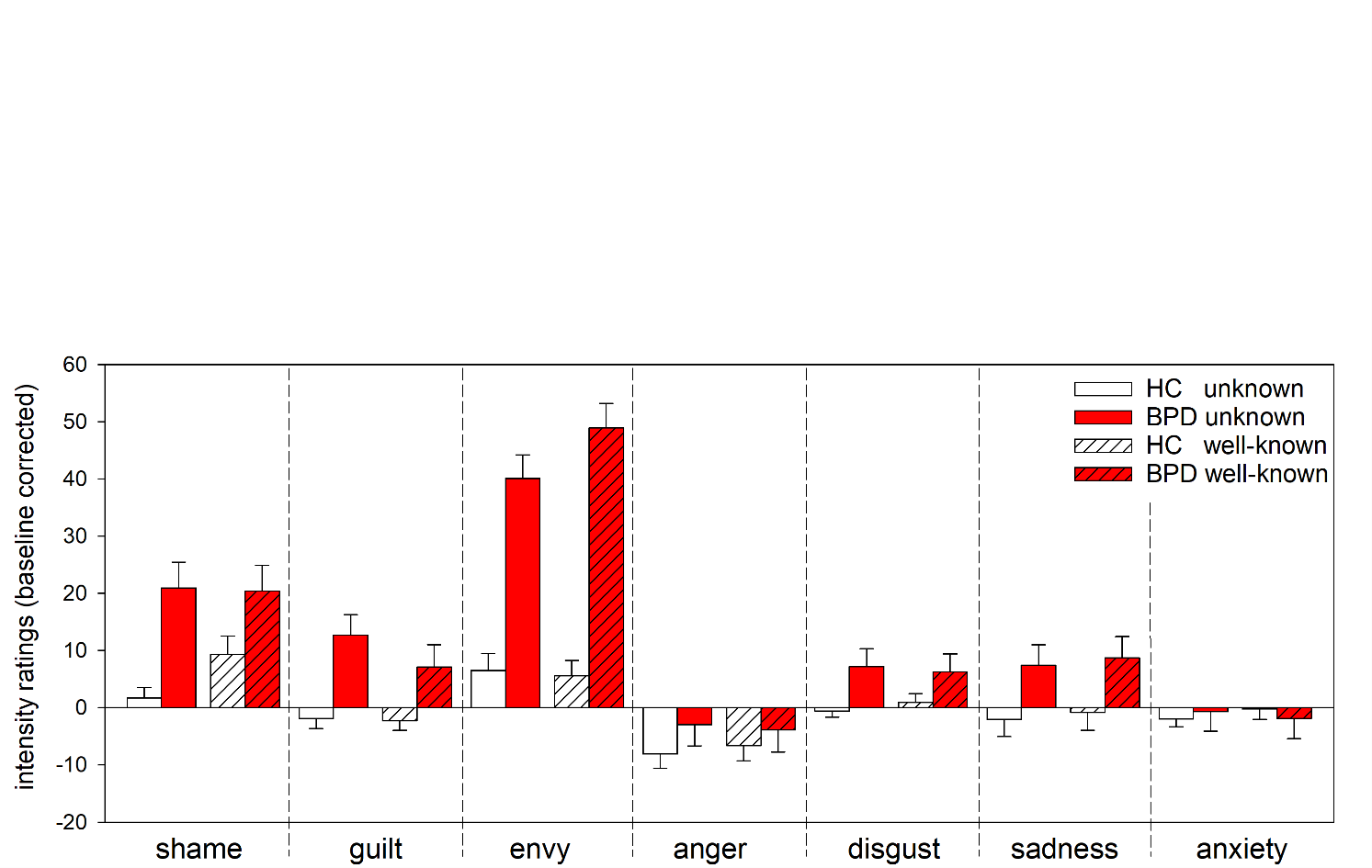
**

*Note.* Mean and standard error of ratings for the single negative emotion categories during the unknown and well-known condition of the experimental task. Ratings scores are corrected for baseline levels of intensity ratings of the different emotion. Scores > 0 indicate an increase in the intensity during the experimental task compared to baseline levels. Exploratory analyses of differences between both conditions revealed higher intensity ratings in the well-known condition compared with the unknown condition for shame in the HC group (MW-U test: *Z* = -3.31, *p*_uncorrected_ < .001, *p*_Bonfcorrected_ *p* = .031) and envy in the BPD group (MW-U test: *Z* = -2.2, *p*_uncorrected_ = .028, p_Bonfcorrected_ *p* =.392).**Table S2.** Results of the 2 × 2 × 7 rank-aligned ANOVA

|  | df_1_, df_2_ | *F* | *p*_GG_ | Cohen’s *f* |
| --- | --- | --- | --- | --- |
| Group | 1, 107 | 67.88 | < .001 | 0.80 |
| Reference | 1, 107 | 89.92 | < .001 | 0.92 |
| Group x Reference | 1, 107 | 41.89 | < .001 | 0.63 |
| Emotions | 6, 642 | 43.61 | < .001 | 0.64 |
| Group * Emotions | 6, 642 | 14.87 | < .001 | 0.37 |
| Reference * Emotions | 6, 642 | 89.34 | < .001 | 0.91 |
| Group * Reference * Emotions | 6, 642 | 52.75 | < .001 | 0.70 |

*Note. p*_GG_: ps corrected according to Greenhouse-Geisser

**Table S3.** *Post-hoc comparisons of intensity ratings in the self and other condition between the HC and BPD group*

|  | other reference | | |  | self reference | | |
| --- | --- | --- | --- | --- | --- | --- | --- |
|  | *Z* | *p* | *p*_Bonf_ |  | *Z* | *p* | *p*_Bonf_ |
| shame | -2.63 | .008 | .119 |  | -4.79 | .000 | < .001 |
| guilt | -3.53 | .000 | .006 |  | -4.98 | .000 | < .001 |
| envy | -6.48 | .000 | < .001 |  | -0.96 | .337 | 1.000 |
| anger | -0.36 | .722 | 1.000 |  | -2.41 | .016 | .221 |
| disgust | -2.15 | .031 | .439 |  | -6.97 | .000 | < .001 |
| sadness | -2.44 | .015 | .206 |  | -4.03 | .000 | < .001 |
| anxiety | -0.02 | .983 | 1.000 |  | -2.18 | .030 | .415 |

*Note. p*_Bonf:_ Bonferroni corrected for 14 pairwise comparisons

**Table S4.** *Exploratory post-hoc comparisons of intensity ratings between the self and other condition in the HC and BPD group*

|  | HC | | |  | BPD | | |
| --- | --- | --- | --- | --- | --- | --- | --- |
|  | *Z* | *p* | *p*_Bonf_ |  | *Z* | *p* | *p*_Bonf_ |
| shame | -4.25 | < .001 | < .001 |  | -6.51 | < .001 | <.001 |
| guilt | -.45 | .654 | 1.000 |  | -3.89 | < .001 | .001 |
| envy | -2.84 | .004 | .063 |  | -6.08 | < .001 | <.001 |
| anger | -2.21 | .027 | .383 |  | -4.07 | < .001 | <.001 |
| disgust | -2.09 | .036 | .509 |  | -6.60 | < .001 | <.001 |
| sadness | -1.95 | .052 | .721 |  | -3.96 | < .001 | .001 |
| anxiety | -1.16 | .247 | 1.000 |  | -3.13 | .002 | .025 |

*Note. p*_Bonf:_ Bonferroni corrected for 14 pairwise comparisons

**Figure S2** Scatterplots for the relationship between shame proneness and ratings of state shame


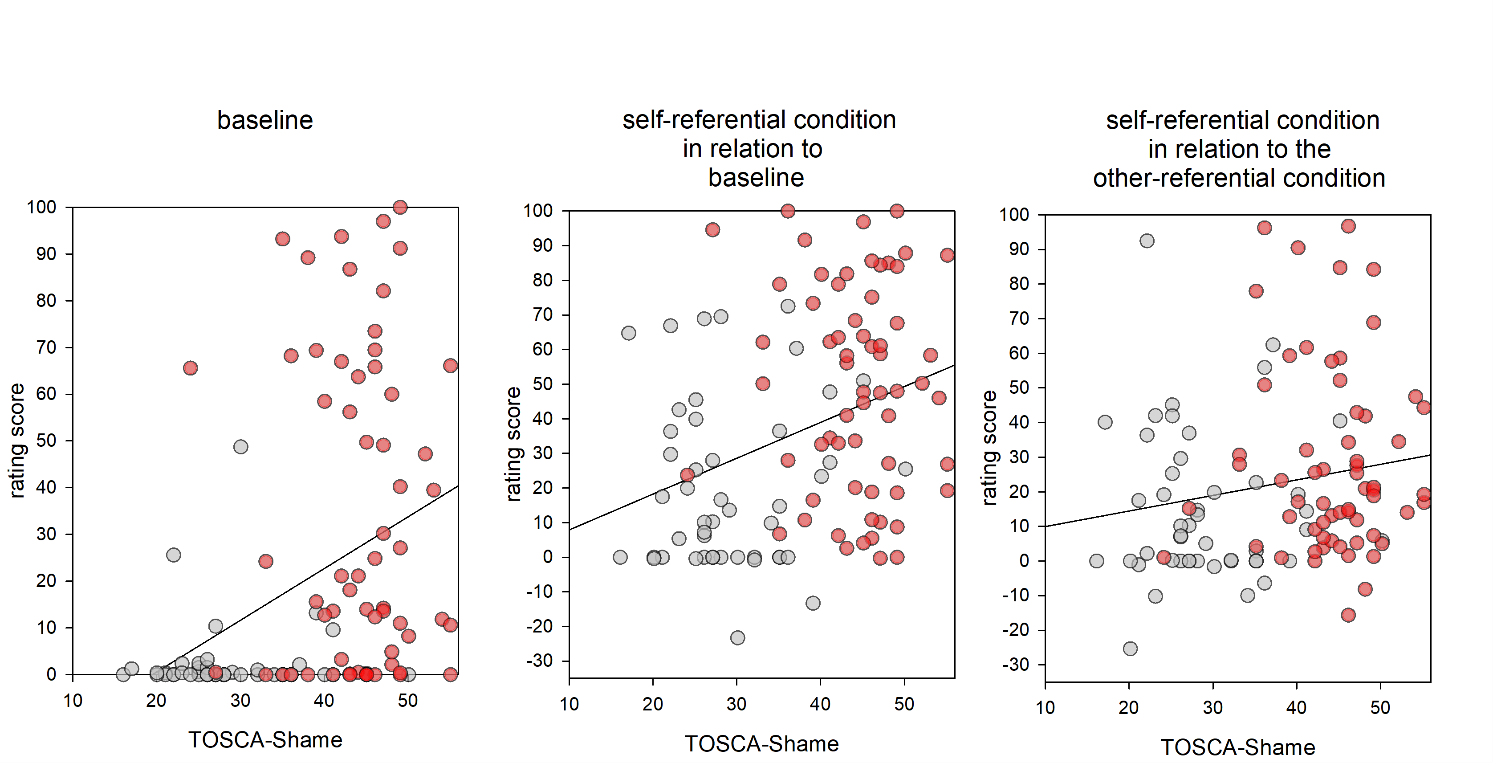


*Note.* TOSCA-shame: subscale of the TOSCA measuring shame proneness; rating score: ratings of shame during the experimental task at baseline (left column), following the self-referential condition in relation to baseline scores (middle column) and in relation to the other-referential condition (right column). Higher rating scores indicate a higher intensity of shame in the self-referential condition in relation to intensity ratings during the baseline and the other-referential condition, respectively. Red circles represent BPD and grey circles represent HC cases.

**Supplementary Material B. Exploratory analyses of positive emotion states**

**Baseline**

At baseline, BPD patients reported lower intensities for positive emotions compared with healthy controls (main effect group: *F*(1,107) = 29.13, *p* < .001, Cohen’s *f* = 0.52). Differences between groups varied between the emotion categories (interaction effect ‘group * emotion’: *F*(3,321) = 3.79, *p* = .019, Cohen’s *f* = 0.19) with the largest effect size for satisfaction (satisfaction: *r* = .43; joy: *r* = .32; pride: *r* = .26; interest: *r* = .22). See Figure S3A, Table S5.

**Experimental task**

BPD patients reported a lower intensity for positive emotions in the self-referential condition compared with the other referential condition than healthy control participants (interaction effect ‘group * reference’: *F*(1,107) = 16.67, *p* < .001, Cohen’s *f* = 0.40). Differences between groups depending on the emotion categories were not confirmed statistically (interaction effect ‘group * reference * emotion’: *F*(3,321) = 2.48, *p*= .069, Cohen’s *f* = 0.15). See Figure S3B, Table S6.

**Figure S3.** Ratings for positive emotions at baseline and during the experimental task

**
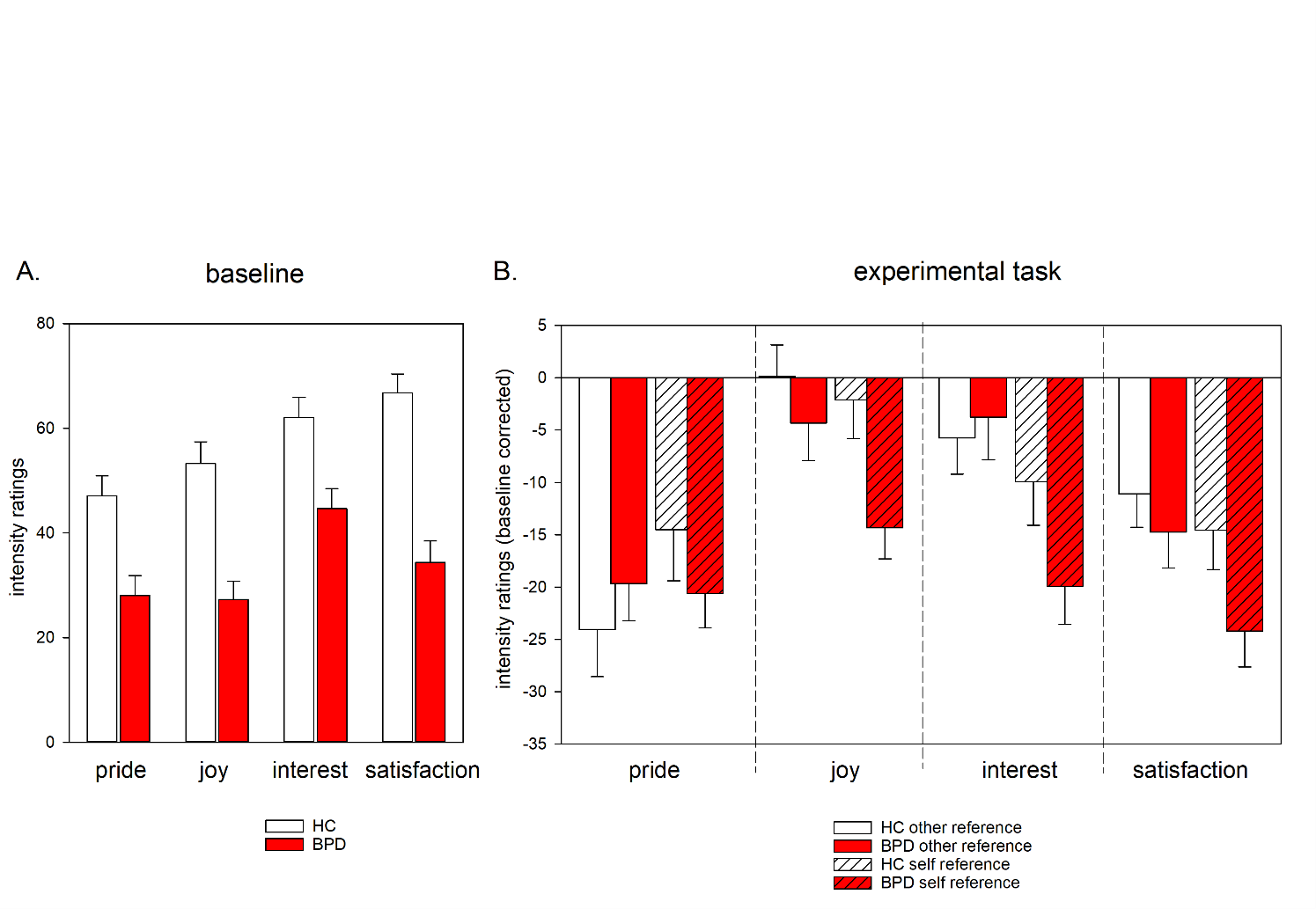
**

*Note.* Mean and standard error of ratings for the different positive emotions (A) during baseline and (B) for the other- and self-referential condition of the experimental task. Please note that rating scores during the experimental task are baseline correct with scores < 0 indicating lower intensity ratings during the experimental task compared to baseline ratings.

**Table S5.** *Results of the 2 ×* *4 rank-aligned ANOVA for positive emotions at baseline*

|  | df_1_, df_2_ | *F* | *p* | Cohen’s *f* |
| --- | --- | --- | --- | --- |
| Group | 1, 107 | 29.13 | < .001 | 0.52 |
| Emotions | 3, 321 | 17.53 | < .001 | 0.40 |
| Group * Emotions | 3, 321 | 3.79 | .019 | 0.19 |

*Note. ps* are corrected according to Greenhouse-Geisser

**Table S6.** *Results of the 2* × *2* × *4 rank-aligned ANOVA for positive emotions during the experimental task*

|  | df_1_, df_2_ | *F* | *p* | Cohen’s *f* |
| --- | --- | --- | --- | --- |
| Group | 1, 107 | 0.75 | .389 | 0.08 |
| Reference | 1, 107 | 14.40 | < .001 | 0.37 |
| Group * Reference | 1, 107 | 16.67 | < .001 | 0.40 |
| Emotions | 3, 321 | 9.72 | < .001 | 0.30 |
| Group * emotions | 3, 321 | 1.04 | .367 | 0.10 |
| Reference * emotions | 3, 321 | 11.35 | < .001 | 0.33 |
| Group * Reference * emotions | 3, 321 | 2.48 | .069 | 0.15 |

*Note. ps* are corrected according to Greenhouse-Geisser
